# Qualitative analysis of the vocabulary used in work logs of a preventive programme for elderly oral function and nutrition

**DOI:** 10.1111/joor.12804

**Published:** 2019-05-08

**Authors:** Kayoko Ito, Ayako Edahiro, Yoshihiko Watanabe, Yuki Ohara, Yoshiko Motohashi, Shiho Morishita, Keiko Motokawa, Yutaka Watanabe, Hirohiko Hirano, Makoto Inoue

**Affiliations:** ^1^ Oral Rehabilitation Niigata University Medical and Dental Hospital Niigata Japan; ^2^ Tokyo Metropolitan Institute of Gerontology Tokyo Japan; ^3^ Faculty of Comprehensive Management Tohoku Fukushi University Sendai Japan; ^4^ Nagoya College of Medical Health and Sports Nagoya Japan; ^5^ Gerodontology Department of Oral Health Science, Faculty of Dental Medicine Hokkaido University Sapporo Japan; ^6^ Division of Dysphagia Rehabilitation Niigata University Graduate School of Medical and Dental Sciences Niigata Japan

**Keywords:** elders, nutrition, oral function, qualitative data

## Abstract

**Background:**

In Japan, day care services for elders include programmes aimed at improving nutrition and oral and motor functions. Few studies have qualitatively assessed these interventions.

**Objective:**

To qualitatively search for the characteristic words used in the work logs of a preventive programme on oral function and nutrition for elders by intervention period and intervention type．

**Methods:**

We included 83 participants (81.3 ± 8.2 years) from four day care services in Japan and divided them into the following groups randomly: those who received oral function intervention only, nutritional intervention only and those who received combined oral function plus nutritional intervention. The interventions were conducted twice per month for 24 months. Data from handwritten work logs were entered into a computer as text files. Monitoring of frequently appearing words, co‐occurrence analysis and cross‐tabulation by intervention period and intervention types was conducted using text mining analysis.

**Results:**

Correspondence analysis revealed that the words used during 1‐6 months and 7‐12 months were similar in participants’ subjective content, and those used in objective content in 13‐18 months and 19‐24 months were similar. These results indicate that subjective improvements increased after 13 months, and it was maintained within 24 months. The combined intervention type is ideal for oral and nutrition problems.

**Conclusion:**

Because this text mining approach revealed the changes in the words used and could be used to monitor any subjective improvement, this approach may help evaluate the effects of preventive care.

## 1 BACKGROUND

The average lifespan of Japanese people has continued to increase; in 2013, the average lifespans of men and women were 80.2 and 86.6 years, respectively. However, the average healthy lifespans of men and women are 71.2 and 74.2 years, respectively.^1^ Thus, overall average lifespans and healthy lifespans differ by approximately 10 years. To extend healthy lifespans, it is critical to prevent individuals from declining to a state in which they require long‐term care.

Preventive care services were introduced in Japan in 2006. A preventive care service comprises three fundamental concepts: improvement of oral function, improvement of nutrition and improvement of motor function. Some services are implemented separately, whereas others are implemented in a combined manner, such as oral function together with nutritional improvement. In implementing the services, the elders are first evaluated for oral function or systemic condition and then trained in accordance with each condition. Oral function programme includes oral health instructions for preventing caries, and periodontitis and oral function training for preventing dysphagia are performed by dental hygienists. Nutrition programmes include nutritional instructions and are conducted by nutritionists. The effects of these programmes are as follows: improvements in swallowing function (assessed using repetitive saliva swallowing test (RSST)), oral diadochokinesis^2^ and lip closure force.^3^ In addition, Morishita et al^4^ reported that a combined intervention programme significantly improved vitality index and pronunciation of the syllable/pa/ in oral diadochokinesis. However, there may be some benefits to preventive care, which are difficult to assess using objective numbers, such as facial expressions, attitudes and emotions.

The text mining approach has been used with various types of qualitative data, such as written records by nurses during disasters,^5^ interview data related to multidisciplinary cooperation among medical professionals^6^ and free‐response content in class assessment surveys.^7^ If the text mining approach is applied to the records of the preventive care programme, other effects, which are difficult to evaluate by an objective numerical value, may become obvious. In addition, by investigating the frequency of occurrence of words, it may be possible to obtain new insights into these programmes. However, no attempt has been made to apply text mining analysis to preventive care programmes. Therefore, we aimed to analyse preventive care service work logs of the intervention study conducted by Morishita et al^4^ to identify the characteristic words used by intervention period and intervention type.

## 2 MATERIALS AND METHODS

### 2.1 Programmes for improving oral function and nutrition

In four elderly day care services in Aichi Prefecture, Japan, 130 elderly were agreed to participate in this study. A total of 35 elderly were excluded because of severe dementia (clinical dementia rating score was 3), poor health condition and hospitalisation as described by Morishita et al^4^ We enrolled 95 participants in our study (35 men and 60 women; mean age 82.7 ± 6.9 years). The participants were randomly divided into three groups using a computer‐generated sequence until the numbers of the participants were equal in all groups: those who received oral function intervention only, those who received nutritional intervention only and those who received a combination of both oral function and nutritional intervention. The interventions for each group were implemented from November 2012 to October 2014. At the end of the study, 83 participants remained. The reasons why the other participants were excluded from the study were as follows: hospitalisation, admission to a nursing home and death. For the 31 participants (15 men and 16 women) in the oral function‐only group, seven dental hygienists provided oral function programme; instructions on oral hygiene, exercise for expression muscles, tongue, salivary glands, swallowing and dysarthria training. For the 23 participants (nine men and 14 women) in the nutrition‐only group, five registered dietitians provided nutrition programme; self‐check of appetite and dehydration, diet balance and weight management. For the last 29 participants (nine men and 20 women) in the combined oral function and nutrition group, both the dental hygienists and the registered dietitians implemented their respective programmes. The same dental hygienists or registered dietitians were in charge of both the single programme and the combined programme. To unify the programme contents, the dental hygienists and registered dietitians received training. The interventions were conducted twice a month over a 24‐month period.

### 2.2 Text mining approach

Text mining techniques were used to analyse the work logs recorded by the seven dental hygienists and five registered dietitians as they conducted their programmes. Text mining is a content analysis method that uses quantitative analysis techniques to sort or analyse qualitative text data.^8^ The use of quantitative techniques to analyse qualitative data prevents arbitrary interpretations on the part of the analyst. The work logs were handwritten by the dental hygienists or registered dietitians just after each programme session for recording and for messages to other performers. It was made for each participant. One log per session consisted of 200‐300 words. They were instructed to describe the records, which were divided into four categories: subjective contents, objective contents, assessments and programme contents. Subjective contents included words used by the elderly individuals themselves, and objective contents included the objective findings of the dental hygienists or registered dietitians. In this study, only subjective and objective contents were used in the analysis.

Except for personal data that would allow identification of individual participants (like names and addresses), the data in all handwritten work logs were entered into the computer as a text file and were then analysed using KH Coder (available from http://khc.sourceforge.net/). 9 We also used ChaSen analysis software for preprocessing and to perform morphological analysis. Compound words such as “full dentures,” “remaining teeth,” “oral cavity” and “Patakara” (a lip trainer) were adopted as force pickup words. At first, frequencies of words were estimated in subjective contents and objective contents. Next, a correspondence analysis was performed to observe the characteristic words by intervention period (1‐6 months, 7‐12 months, 13‐18 months and 19‐24 months) and intervention type (oral function‐only, nutrition‐only and combined). Then, categorisation was performed depending on the type of cluster analysis. Finally, cross‐tabulation was performed by intervention period and intervention type, using the categories. A *P*‐value of <5% was considered significant.

## 3 ETHICAL CONSIDERATIONS

This study was conducted with the approval of the Institutional Review Board of the National Center for Geriatrics and Gerontology (application no. 605). Potential participants received information on the purpose and content of the study before the start of the study; those who provided consent were enrolled as participants. Additionally, all staff who took part in this study provided consent. All data were anonymised and analysed under the condition that individual participants could not be identified.

## 4 RESULTS

### 4.1 Characteristic words and co‐occurrences

In total, 375 238 words (31 189 sentences) were obtained from the handwritten data, with the exclusion of personal information. By categories of records, 138 329 words (20 824 sentences) were used as subjective contents, whereas 115 269 words (20 706 sentences) were used as objective contents.

In the subjective contents, the number of different words, excluding the pure appearance words without duplication, was 6430. The most frequently used word was eat occurring 1320 times, followed by say (590 times) and meal (490 times). The remaining 10 most characteristic words were go (428 times), drink (358 times), night (352 times), denture (346 times), home (312 times), good (296 times) and tongue (289 times). The most co‐occurring word was morning, followed by brushing, eat and meal.

In the objective contents, the number of different words, excluding the pure appearance words without duplication, was 5868. The most frequently used word was tongue coating (901 times), followed by denture (695 times), tongue (666 times), clean (448 times), plaque (427 times), meal (422 times), appearance (409 times), eat (359 times), intervention (351 times) and smile (339 times). The most co‐occurring word was tongue, followed by denture and smile.

### 4.2 Analysis by intervention periods

Table 1 shows the results of the analysis of characteristic words used during the intervention types. The value listed on the right side of each word represents the Jaccard index, rated from 0 to 1. A high Jaccard index indicates a strong characteristic of the word by co‐occurrence analysis. In the subjective contents, the words related to meal and eat were considered as strong characteristic words and indicated that the elders cared about meals. In 1‐6 months and 7‐12 months, denture was on a centred position. After 7 months, the centre words that the evaluator was cared about were tongue coating and plaques in the objective contents.

**Table 1:** Analysis of characteristic words based on intervention period and intervention type

Intervention period								Intervention type					
1‐6 mo		7‐12 mo		13‐18 mo		19‐24 mo		Nutrition‐only		Oral function‐only		Oral function + nutrition	
Subjective session													
Meal	0.049	Say	0.054	Eat	0.101	Eat	0.078	Eat	0.130	Denture	0.068	Eat	0.112
Denture	0.036	Go	0.045	Meal	0.048	Say	0.057	Meal	0.056	Tongue	0.062	Meal	0.056
Before	0.029	Denture	0.04	Night	0.045	Meal	0.044	Drink	0.047	Say	0.062	Say	0.054
Make	0.024	Home	0.034	Go	0.04	Night	0.041	Go	0.042	Brushing	0.041	Go	0.048
Like	0.022	Good	0.034	Drink	0.04	Go	0.038	Good	0.038	Morning	0.034	Night	0.042
Objective session													
Eat	0.042	Tongue coating	0.085	Tongue coating	0.075	Tongue coating	0.067	Meal	0.068	Tongue coating	0.14	Tongue coating	0.078
Say	0.031	Plaque	0.076	Plaque	0.06	Lunch	0.061	Appearance	0.061	Plaque	0.116	Lunch	0.046
Talk	0.025	Denture	0.067	Lunch	0.046	Intervention	0.055	Eat	0.055	Denture	0.109	Appearance	0.043
Little	0.024	Tongue	0.063	Intervention	0.046	Plaque	0.045	Lunch	0.053	Tongue	0.106	Clean	0.043
Before	0.02	Food debris	0.054	Dryness	0.041	Dryness	0.042	Smile	0.05	Food debris	0.081	Smile	0.042

Note

The value listed on the right side of each word represents the Jaccard index. A high Jaccard index indicates strong co‐occurrence relation.

In the correspondence analysis, the appearance patterns of the words in the subjective contents were divided into three phases: ≦12 months, 13‐18 months and 19‐24 months. The words used at 1‐6 months and at 7‐12 months demonstrated an almost similar appearance pattern. The words such as fun and fine were frequently used at 13‐18 months and at 19‐24 months. The objective contents were observed in three phases: 1‐6 months, 7‐12 months and ≧13 months. The words used at 13‐18 months and 19‐24 months demonstrated an almost similar appearance pattern. Of the words, the co‐occurrences for tongue coating and plaque were particularly strong.

Results of the cluster analysis are shown in Table 2. The subjective contents were divided into seven clusters, whereas the objective contents were divided into six clusters. Former clusters were coded “condition,” “intervention,” “oral hygiene,” “daily life,” “meal,” “excretion/sleep” and “medication,” and latter clusters were named “condition,” “oral hygiene,” “tongue coating,” “oral function,” “conversation” and “meal.”

**Table 2:** Categories and included words in subjective contents and objective contents

Contents category	Number of words	Examples of words
Subjective contents		
Condition	7	Condition, body, appetite, change, bad, good, physical condition
Intervention	7	Intervention, instruction, care service, come, enjoy, lunch day care
Oral hygiene	15	Oral hygiene, brushing, teeth, tongue, denture, every day, morning,
Dairy life	27	Go, walk, talk, like, think, do, hear
Meal	11	Meal, eat, make, bread, coffee, milk, daughter
Excretion/sleep	4	Toilet, wake, night, sleep
Medication	3	Medication, tea, drink
Objective contents		
Condition	4	Fine, appearance, good, facial appearance
Oral hygiene	6	Oral, teeth, denture, plaque, remaining teeth
Tongue coating	3	Tongue coating, white, all
Oral function	8	Oral cavity, dryness, buccal, exercise, tongue, power, clean, Ok
Conversation	7	Talk, say, see, voice, conversation, smile, story
Meal	4	Meal, eat, lunch, intake, amount

**Figure 1:**
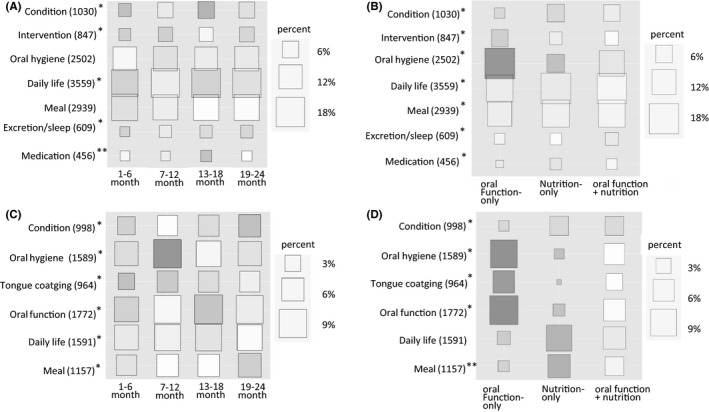
Appearance rate of categories by intervention period (A, C) and intervention type (B, D). Subjective contents were shown in A and B, objective contents were shown in C and D. The larger the square, the higher the appearance rate in the whole. The shading of colour indicates at which intervention period (or type), the appearance rate is high among the same category. The number in y‐axis indicated the appearance times. ^*^ *P* < 0.01; χ2 test. ^**^ *P* < 0.05; χ2 test

The cross‐tabulation analysis of the subjective contents indicated that “daily life” was the most frequent category (3559 times, 17.1%), followed by “meal” (2939 times, 14.1%) and “oral hygiene” (2502 times, 12.0%) (Figure 1A). The appearance frequency of “condition,” “intervention,” “daily life,” “excretion/sleep” and “medication” was significantly different depending on the intervention period. “Condition” and “medication” were observed significantly more in 13‐18 months, and the appearance rates were 6.0% and 2.7%, respectively. In the objective contents, “oral function” was the most frequent category (1772 times, 8.6%), followed by “daily life” (1591 times, 7.7%) and “oral hygiene” (1589 times, 7.7%) (Figure 1C). The appearance rates of all categories were significantly different depending on the intervention period. The appearance rate of “oral hygiene” was higher in 7‐12 months (9.1%) than in other periods (1‐6 months: 6.6%, 13‐18 months: 7.4% and 19‐24 months: 6.7%). “Oral function” had a good appearance rate in 13‐18 months (9.5%), than in 1‐6 months (7.1%), 7‐12 months (8.6%) and 19‐24 months (8.9%).

### 4.3 Analysis by intervention type

Table 1 shows the results of the analysis of characteristic words used during the intervention types. In the combined intervention, characteristic words included words related to diet (like eat and lunch) and words related to oral function training (like massage and stretch). The frequently used words in the oral function‐only intervention group included those related to the parts of the oral cavity (tongue, cheek, etc), those related to oral function training (eg, massage and stretch), and those related to oral hygiene (such as tongue coating and plaque). The frequently used words in the nutrition‐only intervention group included words related to eating or drinking (such as eat, eating and drink) and words describing actions (including speak, say and go).

The correspondence analysis of the subjective contents showed that the characteristic words in the combined intervention appeared at the centre of the oral function‐only intervention and nutrition‐only intervention. The same tendency was observed for objective contents. In the subjective contents, the words demonstrating co‐occurrence in all intervention types were say, meal and eat. However, in the objective contents, none of the words had a co‐occurrence relation to all three interventions, although some words had co‐occurrence relationship between oral function‐only and combined intervention or between nutrition‐only and combined intervention. The words eat, say, meal and lunch had strong co‐occurrences between nutrition‐only and combined interventions, whereas the words tongue coating, denture, plaque and clean revealed strong co‐occurrences between oral function‐only and combined interventions.

The results of the cross‐tabulation analysis of the subjective contents using categories shown in Table 2 are indicated in Figure 1B. The appearance frequencies of all categories were significantly different depending on the intervention type. “Oral hygiene” was more frequently observed in the oral function‐only intervention (18.2%) than in the nutrition‐only (6.4%) and combined intervention (13.1%). “Condition” appeared more frequently in the nutrition‐only and combined intervention “daily life” (18.4% and 17.5%, respectively) and “meal” (14.9% and 14.6%, respectively) appeared more frequently than in the oral function‐only intervention. With regard to the objective contents of the oral function‐only intervention, “oral hygiene” (13.0%), “tongue coating” (8.4%) and “oral function” (14.2%) showed a higher appearance rate than “daily life" (3.1%) and “meal” (2.2%). In the nutrition‐only intervention, the appearance rate of “daily life” (11.6%) and “meal” (9.0%) was higher than that of “oral hygiene” (1.8%), “tongue coating” (0.4%) and “oral function” (2.5%). In the combined intervention, all categories existed at almost the same rate (4.6%‐8.5%).

## 5 DISCUSSION

This is the first study to assess the work logs of preventive care services using text mining approach. The words such as “daily life,” “meal” and “oral hygiene,” which were categorised using cluster analysis, were frequently used throughout the 24‐month period. In other words, elders had cared about “daily life,” “meal” and “oral hygiene” within the two‐year intervention period. On the basis of the correspondence analysis of the objective contents, the focus of the staff was moved from “oral hygiene” in 7‐12 months to “oral function” in 13‐18 months. In the early stages, the staff focused first on “oral hygiene.” After improvements were observed, they focused on “oral function.” The correspondence analysis also revealed that the words used in 1‐6 months and 7‐12 months were similar in subjective contents. In 13‐18 months and 19‐24 months, the words that indicated a good condition such as fine and fun were observed. In addition, the words that appeared in 13‐18 months and 19‐24 months were similar in objective contents. These results may indicate that subjective improvements had increased after 13 months and hence another set of interventions may be started and maintained within 24 months. In this study, we divided the intervention period into six months based on a previous study, which had reported improvements in bite force, RSST and salivary flow rate after a six‐month preventive care programme.^10^ However, no study has reported on the suitable intervention period for such analyses. To monitor the changes over time, the data should be collected and analysed on a monthly basis.

Analysis of characteristic words revealed that the most frequently used word was eat, followed by say and meal in the subjective contents, and tongue coating, denture and tongue in the objective contents. There were eight types of words that were related to dentures: full dentures, full false teeth, dentures, partial dentures, partial false teeth, false teeth, FD and PD. In a study of comprehension of dentistry terms conducted among care workers, 100% of the participants understood the term dentures, whereas only 80% of the participants understood the terms full dentures and partial dentures.^11^ The use of technical terms will not become a problem if the preventive care programme is conducted only by one discipline. However, when the care becomes multidisciplinary, replacing technical terms with lay expressions may be important for proper sharing of information.

A correspondence analysis by intervention type showed that the characteristic words in the combined intervention appeared at the centre of the oral function‐only intervention and nutrition‐only intervention. A cross‐tabulation analysis revealed that the words used in the oral function‐only or nutrition‐only intervention were biased; however, the words were used in a well‐balanced manner in the combined intervention. When evaluated using objective numbers, a combined intervention programme significantly improved vitality index and pronunciation of the syllable/pa/ in oral diadochokinesis.^4^ In addition, in the study conducted in 2010, we combined a programme for improving motor function and improving nutrition with a programme for improving oral function. Consequently, compared with unidisciplinary programmes, a higher percentage of participants in the combined oral function plus nutrition programme had a lower need for nursing care, as well as a reduced risk of health decline to a level requiring care (ie, reduced risk of falls, fractures, aspiration pneumonia and the like), suggesting that the combined programme yielded a greater preventive care effect.^12^ Therefore, there is a possibility that the combination intervention type is ideal to cover oral and nutrition problems.

This study had some limitations. The number of participants who could not finish the 24‐month continued intervention was higher in the nutrition‐only intervention type. Furthermore, the bias in the number of participants led to the bias in the number of records and thus influenced the results of the analysis. Another limitation was that the staff involved in the single and combined intervention type overlapped. In the future, the analysis should be conducted on the basis of the difference between occupation type and intervention form. To make the difference in occupation clear, the single intervention and combined intervention should be performed by different staffs without concurrent operation.

## 6 CONCLUSION

The text mining approach was applied to the work logs of preventive care services. It was suggested that the elders cared about “daily life,” “meal” and “oral hygiene.” A correspondence analysis revealed that subjective improvements had increased after 13 months and hence another set of interventions must be performed and this condition must be maintained within 24 months. In addition, there was a possibility that the combination intervention type was ideal to cover oral and nutrition problems. Because this text mining approach revealed changes in the word used and showed subjective improvements, it may contribute to the evaluation of the effect of preventive care.

## References

[1] Ministry of Health LaWJ . The second term of National Health Promotion Movement in the twenty first century. 2015 http://wwwmhlwgojp/stf/seisakunitsuite/bunya/kenkou_iryou/kenkou/kenkounippon21html. Accessed September 23, 2017.

[2] Sakayori T , Maki Y , Hirata S , Okada M , Ishii T . Evaluation of a Japanese “prevention of long‐term care” project for the improvement in oral function in the high‐risk elderly. Geriatr Gerontol Int. 2013;13:451‐457. 22963330 10.1111/j.1447-0594.2012.00930.x

[3] Ooka T , Haino T , Hironaka S , Mukai Y . The effect of daily oral function training in the elderly. J Dent Hlth. 2008;58(2):88‐94.

[4] Morishita S , Watanabe Y , Hirano H , et al. A Long‐term intervention research for the effect of nutrition and oral function improvement service at the daycare facilities. JJSDH. 2017;12(1):36‐46. (in Japanese).

[5] Goto A , Rudd RE , Lai AY , et al. Leveraging public health nurses for disaster risk communication in Fukushima City: a qualitative analysis of nurses’ written records of parenting counseling and peer discussions. BMC Health Serv Res. 2014;14:129.24642079

[6] Miki S . Medical professionals’ cooperative structure and its developmental requirements for a community nutrition support team. J Kyorin Medical Society. 2016;47(2):91‐112. (in Japanese).

[7] Nakagawa K , Yamada K , Asakawa Y , Yamaguchi H . What kinds of impressions did physical therapy students receive through participation in off‐campus classes? an analysis using text‐mining. J Phys Ther Sci. 2012;24:1063‐1068. (in Japanese).

[8] Shinozaki M , Asakawa Y , Ohashi Y . Effects of continuous education using problem‐based learning tutorial systems: a quantitative text analysis of students’ questionnaire responses. J Phys Ther Sci. 2016;31(6):819‐827. (in Japanese).

[9] Takiguchi T . A review of oral epidemiological statistics part X:a trial to adopt quantitative statistical evaluations for results of qualitative analysis using free description type questionnaires – synergy analyses using KH Coder and statistics R. Health Sci Health Care. 2016;16:4‐28.

[10] Ibayashi H , Fujino Y , Pham TM , Matsuda S . Intervention study of exercise program for oral function in healthy elderly people. Tohoku J Exp Med. 2008;215:237‐245. 18648184 10.1620/tjem.215.237

[11] Minami C . Oral health activities of a dental hygienist in a nursing care facility for the elderly:collaboration with professionals from different disciplines. Jpn J Geriatr. 2009;24:389‐392. (in Japanese).

[12] Watanabe Y , Iida R , Ikezoe S , Ito K , Iwasa Y , Ueda K , et al. Comprehensive research project on promotion of oral function improvement services in preventive benefits and long‐term care benefits, a report of a grant for promoting elderly health care services. Labor and Welfare: Ministry of Health;2011. (in Japanese).

